# Health Implications of PAH Release from Coated Cast Iron Drinking Water Distribution Systems in the Netherlands

**DOI:** 10.1289/ehp.1205220

**Published:** 2013-02-19

**Authors:** E.J. Mirjam Blokker, Bianca M. van de Ven, Cindy M. de Jongh, P.G.G. (Nellie) Slaats

**Affiliations:** 1 KWR Watercycle Research Institute, Nieuwegein, the Netherlands; 2 RIVM (National Institute for Public Health and the Environment), Bilthoven, the Netherlands

**Keywords:** bitumen, cast iron, coal tar, drinking water quality, health risk assessment

## Abstract

Background: Coal tar and bitumen have been historically used to coat the insides of cast iron drinking water mains. Polycyclic aromatic hydrocarbons (PAHs) may leach from these coatings into the drinking water and form a potential health risk for humans.

Objective: We estimated the potential human cancer risk from PAHs in coated cast iron water mains.

Method: In a Dutch nationwide study, we collected drinking water samples at 120 locations over a period of 17 days under various operational conditions, such as undisturbed operation, during flushing of pipes, and after a mains repair, and analyzed these samples for PAHs. We then estimated the health risk associated with an exposure scenario over a lifetime.

Results: During flushing, PAH levels frequently exceeded drinking water quality standards; after flushing, these levels dropped rapidly. After the repair of cast iron water mains, PAH levels exceeded the drinking water standards for up to 40 days in some locations.

Conclusions: The estimated margin of exposure for PAH exposure through drinking water was > 10,000 for all 120 measurement locations, which suggests that PAH exposure through drinking water is of low concern for consumer health. However, factors that differ among water systems, such as the use of chlorination for disinfection, may influence PAH levels in other locations.

Several polycyclic aromatic hydrocarbons (PAHs) have been associated with the development of a wide range of cancers [Boffetta et al. 1997; World Health Organization (WHO) 2003]. For this reason, many countries have limits in their drinking water standards for a number of PAHs (Drinking Water Decree 2011; European Communities 1998; U.S. Environmental Protection Agency 2009). In the Netherlands, only 3 of > 1,000 routine samples collected between 2006 and 2009 exceeded drinking water quality standards for PAHs (Versteegh and Dik 2007, 2008, 2009, 2010). In spring 2009, a Dutch water company received complaints about the odor and taste of drinking water after cast iron water mains were flushed (Blokker et al. 2010). After analyzing water samples, the water company concluded that the complaints may have been a result of PAHs in the drinking water, which were assumed to have originated from the bitumen coating in the cast iron mains. Because PAHs are seldom found in drinking water, this raised questions about the frequency of increased PAH levels, the circumstances in which this can occur, and the potential implications for human health.

Cast iron water mains are susceptible to corrosion (van den Hoven and van Eekeren 1988), and the resulting corrosion products can lead to a reduction in drinking water quality and a potential increase in customer complaints (Slaats and Rosenthal 2001). Historically, substances such as coal tar or bitumen were used to coat the insides of iron water pipes, which reduces corrosion by forming a water-resistant barrier on the pipe walls (Maier 1998; Miller et al. 1982; van den Hoven and van Eekeren 1988). This practice was used from around 1900 until it was phased out in the 1970s. Since then, metal pipes have been coated with cement linings.

Both coal tar and bitumen are highly viscous liquids that include a complex mixture of polycyclic aromatic compounds (Zander 1995). The exact composition of coal tar and bitumen can vary depending on the source of coal or petroleum and the processing techniques used to produce them (Read and Whiteoak 2003). The relatively hydrophobic PAHs have a high affinity for sorption onto the surface of particulate matter (De Maagd et al. 1998; Jonker and Koelmans 2002) and thus are usually found in the natural environment in soils or sediment samples, rather than in water or air (Karlsson and Viklander 2008; WHO 2003). Under favorable conditions, biofilms can form in water distribution networks, such as areas with an available nutrient source, stable flow conditions, and a suitable surface or substrate on which to grow (O’Toole et al. 2000; van der Kooij 2002). Maier et al. (2000b) demonstrated that coal tar coatings in water mains support and promote the growth of microbiological colonies to a greater degree than do uncoated stainless steel because the coal tar linings act as a substrate and nutrient source for the development and support of biofilm.

Although the purpose of coating cast iron water mains is to protect the cast iron from corrosion, the linings themselves can deteriorate over time. As a result, PAHs can leach into water during low-flow conditions when the water stays in the mains for longer periods (Alben 1980; Bowen et al. 2000; Brandt and de Groot 2001; Maier 1998). In addition, soft, corrosive water can degrade coal tar and bitumen linings, possibly leading to the release of PAH-containing particulate matter into the water (WHO 2003). Maier (1998) concluded that diffusion from the linings alone probably does not account for PAH levels in drinking water, and suggested that leaching could increase when fresh fracture surfaces are created as a result of a water main breakage or invasive maintenance. Damage to coal tar and bitumen linings during invasive maintenance could also cause the release of particulate matter that contains PAHs (Maier 1998; WHO 2003). Oxidation—due to the presence of dissolved oxygen or chlorine or exposure to ultraviolet radiation—is thought to play a part in the hardening of bitumen, which makes the coating more susceptible to damage (Read and Whiteoak 2003), especially during invasive interventions in the network. Hydraulic disturbances, such as flow changes or pressure fluctuations, could lead to the resuspension of accumulated sediment (Vreeburg 2010), possibly resulting in elevated PAH levels in drinking water (Maier 1998; Maier et al. 2000b). Although biofilm may prevent the leaching of PAHs from the coatings into the water supply (Maier 1998; Maier et al. 2000a, 2000b), increased PAH levels have been observed in water supplied through coal tar–lined water mains following the use of chlorine as a disinfectant (Maier D et al. 1997; Maier M et al. 2000b), presumably because of the breakdown of protective biofilm. Chlorination, deoxygenation, removal of a nutrient source, hydraulic disturbances, and low temperatures can all result in biofilm disintegration.

To summarize, PAHs may leach from bitumen coatings into drinking water, and the erosion of bitumen coatings may release particles that contain PAHs. PAHs may be increased when PAH-contaminated sediment particles are resuspended. Thus, PAHs might be expected to increase during periods of stagnant water (resulting in increased leaching due to long exposure times), during flushing of pipes (because of sediment resuspension and biofilm destruction), and during maintenance (invasive work on pipes).

The literature on PAHs in drinking water distribution systems is limited, and there is little quantitative information on the presence of PAHs in drinking water. Therefore, in 2010 we carried out an extensive research project in collaboration with all Dutch water companies, which serve a total population of almost 17 million people. PAHs were measured in samples collected at 120 locations under different operational conditions to identify the conditions associated with increased PAHs in drinking water, and to estimate the potential human health impact of the resulting PAH exposures.

## Materials and Methods

*Measurement locations*. The Dutch drinking water distribution system has a total length of 112,000 km, of which approximately 10% is cast iron mains installed before 1980. We selected 120 measurement locations throughout the Netherlands with cast iron and steel mains 80–120 mm in diameter that were likely to be coated with coal tar or bitumen because they were constructed before 1980. This resulted in approximately 1 sampling location per 100 km of mains. All 10 Dutch water companies participated in the study. The number of measurement locations for each water company was determined based on the length of suspect mains in their networks. We made no further distinctions between water companies.

*Measurement protocol*. Drinking water companies collected water samples from cast iron mains in their own districts under well-defined conditions: during undisturbed operations, during and directly after flushing of pipes, and after the removal of a section of pipe and repair of the water main (Table 1). Two of three samples collected during undisturbed operation were taken with some valves closed to ensure a single feed and a well-defined flow direction. Samples obtained during flushing were taken directly from water hydrants. All other samples were obtained from taps located on the distribution mains that were installed by the water companies prior to the measurement period. For practical reasons, we did not collect residential tap water samples because access could not be guaranteed at all sampling times.

**Table 1:** Sampling times, sample conditions, and estimated exposure durations.

Day	Sample	Condition at sample collection	Exposure duration (days)
1	Undisturbed 1	At tap during undisturbed normal operation
3	Undisturbed 2	At tap during undisturbed operation on single feed	
8	Low-flush	At hydrant during low-velocity flush (0.35 m/sec), 15 sec after opening hydrant	1
8	High-flush	At hydrant during high velocity flush (1.00 m/sec), 15 sec after opening hydrant	1
8	After-flush	At tap 15 min after hydrant was closed during undisturbed operation (on single feed)	2
10	Undisturbed 3	At tap during undisturbed operation (on single feed)	319a
15	Repair 1	At tap during undisturbed operation (on single feed) 2–4 hr after pipe was closed for the removal and repair of a piece of cast iron main	2
17	Repair 2	At tap 2 days after pipe was closed during undisturbed operation (on single feed)	40
aWe assumed that the highest PAH concentration of the three samples collected during undisturbed operation was representative of PAH levels for the rest of the year (319 days).			

The water companies collected samples from each measurement location eight times over a 2.5-week period: six times at a tap and twice at a hydrant (Table 1). For low- and high-flush samples collected from hydrants, flushing flows corresponding to estimated velocities of 0.35 m/sec and 1.0 m/sec, respectively, were maintained using a specially constructed caliber plate or by measuring the flushing flow. The flush samples were not collected as part of a cleaning program designed to remove all particles in a systematic way (Vreeburg 2010). During the flushing actions (day 8), the flow (in cubic meters per hour) and pressures were recorded. We calculated the actual flushing flow velocities from the measured flow, pressure, and the effective diameter and wall roughness as determined from the removed pieces of the mains. On day 15, the water companies removed one 10–50 cm segment of the cast iron main upstream of each sampling location, and subsequently repaired the main by replacing the removed segment with PVC (polyvinyl chloride) pipe. The water companies collected repeat samples at the locations where the drinking water standards for the sum of 10 PAHs (Σ10PAH: anthracene, benz[*a*]anthracene, benzo[*b*]fluoroanthene, benzo[*ghi*]perylene, benzo[*k*]fluoroanthene, chrysene, fluoroanthene, indeno[1,2,3-*cd*]pyrene, phenanthrene, and pyrene; Table 2) were exceeded until no exceedances were found. This was performed according to the Drinking Water Decree (2011) and was not part of our measurement protocol. Thus, the time between the day-17 sample and the repeat sample varied between water companies. We did use the information to determine the duration of PAH exceedances.

**Table 2:** PAH exceedances of the Dutch Drinking Water Decree (2011) in samples, based on the maximum concentrations measured in any sample of a given type (120 samples per type).

Sample	Percent of samples exceeding standarda		Σ8PAH		
	Σ10PAH	BaP	Max conc (µg/L)	Max oral exposure (µg/kg bw/day)	MOE
Undisturbed 1	0	0	0.003	0.0001	4,900,000
Undisturbed 2	0	0	0.001	0.00003	14,700,000
Low-flushb	60	49	19	0.62	794
High-flushb	60	61	22	0.75	656
After-flush	29	24	2.1	0.069	7,067
Undisturbed 3	2	0	0.020	0.0007	735,000
Repair 1	40	6	2.0	0.069	7,313
Repair 2	31	2	0.23	0.0077	63,913
Abbreviations: Σ10PAH, sum anthracene, benz[a]anthracene, benzo[b]fluoroanthene, benzo[ghi]perylene, benzo[k]fluoroanthene, chrysene, fluoroanthene, indeno[1,2,3-cd]pyrene, phenanthrene, and pyrene; Σ8PAH, sum of benzo[a]pyrene (BaP), chrysene, benz[a]anthracene, benzo[b]fluoranthene, benzo[k]fluoranthene, benzo[ghi]perylene, dibenz[ah]anthracene, and indeno[1,2,3-cd]pyrene); bw, body weight; conc, concentration; Max, maximum; MOE, margin of exposure. aDrinking water quality standards are 0.1 µg/L for Σ10PAH and 0.01 µg/L for BaP.bSamples were collected at hydrant.					

Each water company sent their samples to one of the five accredited laboratories in the Netherlands. These laboratories analyzed each sample for 16 U.S. Environmental Protection Agency priority PAHs (Agency for Toxic Substances and Disease Registry 1995), pH, total organic carbon, electric conductivity, temperature, and turbidity. Four laboratories identified the PAHs using high-performance liquid chromatography with fluorescence detection after solid phase extraction based on NEN-ISO 7981-2:2005 [Netherlands Standardization Institute and International Organization for Standardization (NEN-ISO) 2005]; the fifth laboratory used gas chromatography–mass spectrometry after liquid–liquid extraction using its own internal methodology for the analysis of semivolatile compounds. The detection limits varied from 0.005 μg/L to 0.05 μg/L between laboratories and between PAHs. Round-robin tests were conducted in 2010 to determine the interlaboratory variability in PAH measurements: The resulting average SD was 0.017 μg/L (Asgadaouan 2010).

For each of the 120 cast iron segments that were removed from the sampling locations, we measured the inside diameter, wall thickness, wall roughness (in millimeters), and effective remaining diameter with calipers and visually assessed the coating. When the visual assessment was inconclusive (in approximately 35% of samples), we used a burner to heat the main and performed a scent-based assessment to determine the presence and type of coating. We categorized the type of coating [no coating, bitumen coating, coal tar coating, or unidentified (i.e., there was a coating, but there was no certainty of it being coal tar or bitumen)]; the thickness of the coating [thin (< 1 mm) or thick (≥ 1 mm)]; and the coverage of the main [none, less than one-half, more than one-half, and full (0%, > 0% to 50%, > 50% to < 100%, or 100%)]. The drinking water companies provided information on the years of installation. We examined whether the year of installation was indicative for the coating coverage.

*Health risk assessment*. Cancer is the main outcome of concern for PAH exposure. Risk assessment followed the method used by the European Food Safety Authority (EFSA) to evaluate the risk of PAH contamination in food (EFSA 2008), which is based on the margin-of-exposure (MOE) approach recommended for genotoxic carcinogens in food (EFSA 2005). The MOE is the ratio between a predefined reference dose and the estimated human intake. Usually the reference dose is the BMDL_10_ (benchmark dose, lower confidence limit), that is, the lower 95% confidence limit estimated for the dose that causes no more than a 10% increase in cancer incidence in rodents. The EFSA (2005) is of the view that, in general, an MOE of ≥ 10,000, if it is based on the BMDL_10_ from an animal study, would be of “low concern from a public health point of view and might be considered as a low priority for risk management actions.” The EFSA (2005) concluded that the sum of eight PAHs (Σ8PAH; benzo[*a*]pyrene (BaP), chrysene, benz[*a*]anthracene, benzo[*b*]fluoranthene, benzo[*k*]fluoranthene, benzo[*ghi*]perylene, dibenz[*ah*]anthracene, and indeno[1,2,3-*cd*]pyrene) can be used as indicators of the carcinogenic potency of PAH mixtures. The EFSA also uses the sum of four PAHs (Σ4PAH; BaP, chrysene, benz[*a*]anthracene, benzo[*b*]fluoranthene), the sum of two PAHs (Σ2PAH; BaP and chrysene), and the concentration of BaP alone, to assess carcinogenicity (EFSA 2008).

For each water sample, we calculated the concentration of each of the 16 individual PAHs as well as Σ2PAH, Σ4PAH, and Σ8PAH. For each study location, we multiplied the PAH concentrations in each sample by a worst-case estimate of the duration of exposure to that sample (Table 1) and summed the results to derive an estimate of the average exposure per year in each location; for our analysis, we assumed that this exposure level would be constant over an individual’s lifetime. We assumed that flushing and mains repair would occur once per year, and the duration of exposure to measured concentrations during flushing or repair were determined by the maximum duration of PAH exceedances in this study. The number of mains repairs in cast iron mains was reported by the Dutch water companies as 0.05–0.42/km/year. The highest reported values included mains repairs under pressure (i.e., without taking out a part of the main). With 112,000 km of cast iron mains and 7.3 million household connections, the average exposure to cast iron mains repairs in the Netherlands is well below once per year. We found a PAH exceedance on day 57 (40 days after sample collection for repair 2), which indicates the duration of the PAH exceedance. Although consumers generally do not drink water obtained from hydrants during flushing, we used measured values in these samples to estimate exposures during flushing as a worst-case scenario. For oral intake, we assumed an average body weight of 60 kg and water intake of 2 L/day (WHO 2011).

We calculated the MOEs for each of the summed PAH combinations by dividing the specific BMDL_10_ values [0.07, 0.17, 0.34, and 0.49 mg/kg body weight (bw)/day for BaP, Σ2PAH, Σ4PAH, and Σ8PAH, respectively] derived by the EFSA (2008) by the estimated average annual exposure across all 120 locations. To evaluate noncarcinogenic effects of PAHs, we compared the estimated daily intake of the sum of all of the PAHs (Σ16PAH) with the tolerable daily intake (TDI) of 30 µg/kg bw/day derived by Baars et al. (2001) for PAHs containing < 17 hydrogen atoms per molecule (benzo[*g,h,i*]perylene), which is more stringent than the TDI of 40 μg/kg bw/day for noncarcinogenic PAHs with 17–35 hydrogen atoms (anthracene, fluorine, naphthalene, and phenanthrene). The TDI represents an estimate of the daily intake level to which humans can be exposed during their entire lifetime without causing adverse health effects.

In addition to estimating oral intake, we estimated inhalation exposure during showering using ConsExpo software (RIVM 2010), taking into consideration both aerosol formation and evaporation of PAHs. We did not estimate dermal exposure during bathing or showering because *a*) data on dermal absorption of PAHs from aqueous matrices are scarce, *b*) absorption of PAHs is highly dependent on the vehicle used in the tests, and *c*) available information suggests that exposure from dermal absorption would be negligible compared with oral ingestion from drinking water. Specifically, Moody and Chu (1995) examined dermal absorption of ^14^C-labeled phenanthrene *in vitro* in human skin and observed 24% absorption after exposure of 0.64 cm^2^ skin for 24 hr to a 0.1 mL solution of 1 µg/mL phenanthrene. From this, an equilibrium constant in terms of partial pressure (*K*_p_) was determined (1.6 × 10^–3^ cm/hr) (Moody and Chu 1995). On the basis of a maximum phenanthrene concentration of 6 μg/L in drinking water (Table 2) and a surface body area of 18,000 cm^2^, we calculated that 30 min of daily bathing or showering would result in a dermally absorbed phenanthrene dose of only 86 ng/person/day, compared with our estimated 12,000 ng/person/day from daily oral ingestion of 2 L of drinking water. Data from other studies indicate that dermal absorption of other PAHs is not significantly higher than absorption of phenanthrene (Moody and Chu 1995; Sartorelli et al. 1999; Van Rooij et al. 1995).

## Results and Discussion

*Water PAH levels and different measurement circumstances*. We observed each of the 16 PAHs in at least 1 of the 960 samples (8 samples for each of 120 sampling locations). Of the PAHs measured, naphthalene, phenanthrene, pyrene, and fluoranthene were the most prevalent. Fluoranthene was detected more often than any other PAH in the low- and high-flush samples, and phenanthrene was detected more often in samples collected after main repair (repair 1 and repair 2) (Blokker et al. 2010).

**Figure 1:**
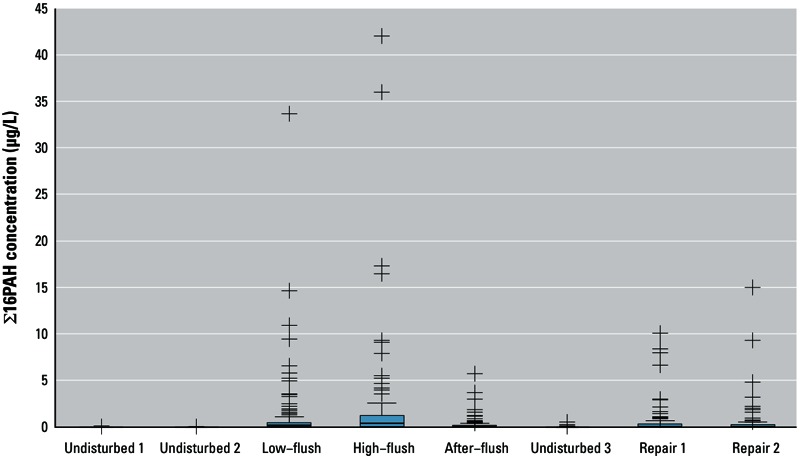
Box and whisker plot of Σ16PAH in each sample type (*n* = 120 for each). Boxes represent the 25th–75th percentiles; horizontal lines within boxes represent medians; whiskers extend 1.5 times the length of the interquartile range above and below the 75th and 25th percentiles; and outliers are represented as “+” symbols.

During normal operation (undisturbed samples 1, 2, and 3), PAHs were undetectable or were present only at very low levels (Figure 1), which is consistent with expectations given that PAH drinking water standards are rarely exceeded in the Netherlands. This suggests that leaching of PAHs from bitumen coatings is not likely to lead to elevated PAH levels in drinking water during normal operation.

Flushing samples collected at the hydrants contained the highest PAH levels (Figure 1), which suggests that disturbance of sediment in cast iron mains may lead to high PAH levels, and that the PAHs measured in these samples are associated with particles in the distribution network. PAH levels were also elevated in samples collected after main repair, which supports the hypothesis that erosion of coating particles—in this case due to mains breakage—leads to increased PAH levels. The duration of increased PAH levels after repair varied: Of the 48 repair 1 samples with PAH levels that exceeded the drinking water quality standard of 0.1 µg/L for Σ10PAH, PAHs were undetectable in 13 samples 2 days later (repair 2), in 28 samples 30–40 days after repair, and in 3 samples taken 42, 44, and 57 days after repair. Of the 48 locations with elevated PAHs after repair, 5 were not resampled after the repair 2 sample was collected.

Because PAHs are hydrophobic, we expected PAHs to adsorb to sediment particles; high PAH levels in the flushing samples were consistent with this expectation. However, PAH levels were not correlated with turbidity (an indication of the amount of suspended particles in the sample) or with estimated flushing velocities (which are associated with sediment resuspension) (data not shown). In addition, PAH levels were not correlated with total organic carbon, pH, electric conductivity, or temperature (*R*^2^ < 0.2; data not shown).

**Figure 2:**
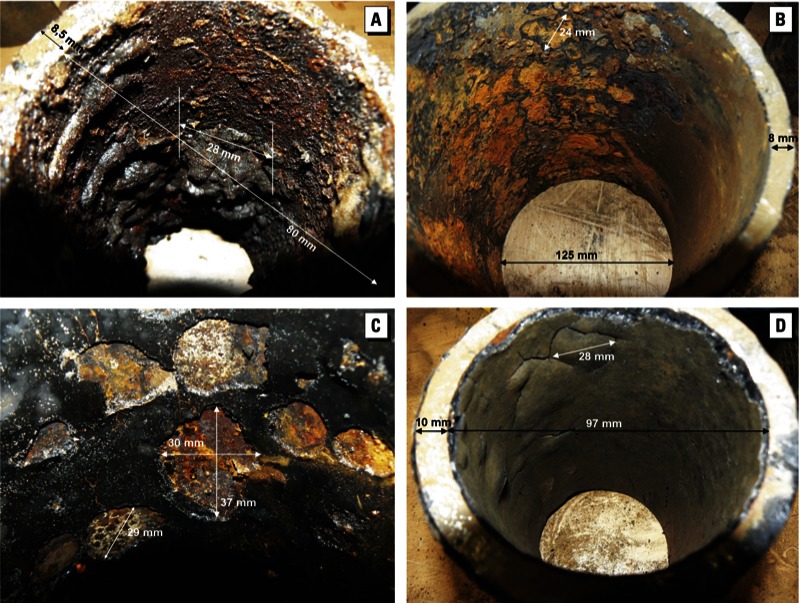
Photographs showing coating of of water main segments from (*A*) location 104: 0% coal tar coating; (*B*) location 41: > 0 to 50% bitumen coating (thin); (*C*) location 83: > 50 to < 100% bitumen coating (thick); and (*D*) location 34: 100% bitumen coating (thick).

**Figure 3:**
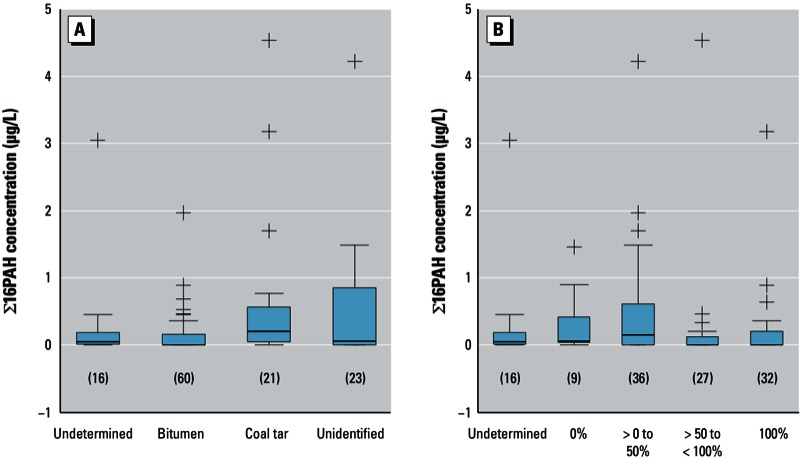
Box and whisker plot of PAH concentrations in repair 1 samples (collected 2–4 hr after a water main segment was removed upstream of the sampling location) by (*A*) type of coating and (*B*) percent of coating coverage. Numbers in parentheses indicate the number of samples. Boxes represent the 25th–75th percentiles; horizontal lines within boxes represent medians; whiskers extend 1.5 times the length of the interquartile range above and below the 75th and 25th percentiles; and outliers are represented as “+” symbols.

*Relationship between PAH and coating*. Of the 120 inspected water main segments, 104 had a coating (Table 3). For the remaining 16 segments, we could not determine whether the main was coated at the time of installation even though the scent-based assessment we used can often reveal whether the main originally did have a coating even if no coating was currently present. Of the 104 pipe segments with an established coating, we assessed the coating type and coverage (Table 3). We found bitumen, coal tar, and some unidentified coating types of varying thickness and coverage (Figure 2). In several of the 16 locations with undetermined presence of a coating in the pipes, PAHs were detectable during flushing (data not shown) and after mains repair (Figure 3). In these cases, it is possible the rest of the cast iron main may have been coated even though the segment that was removed had no coating. Alternatively, the observed PAHs may have originated from another part of the network.

**Table 3:** Coating characteristics of cast iron pipe segments.

Coating characteristic		No. of pipes	Percent of pipesa
Coated	Yes	104	87
	Undetermined	16	13
Coating type	Bitumen	60	58
	Coal tar	21	20
	Unidentified	23	22
Thickness	Thin	70	67
	Thick	34	33
Coverage (%)	0	9	9
	> 0 to 50	36	35
	> 50 to < 100	27	27
	100	32	31
aFor “coated” and “undertermined,” values are percentages of all samples (n = 120); for all others, values are percentages of coated pipes only (n = 104).			

We also observed differences in PAH levels between the types of coatings (Figure 3). Coal tar coatings were associated with higher mean PAH levels than were bitumen coatings (0.63 µg/L; 95% CI: 0.11, 1.16 and 0.13 µg/L; 95% CI: 0.05, 0.21, respectively). Because water companies do not know which of their cast iron mains have bitumen coatings and which have coal tar coatings, the effect of coating type is of no practical value.

Coated main segments with > 0–50% coverage had the highest mean PAH levels of the water samples taken immediately after the segment was removed (Figure 3), although one-way analysis of variance showed no differences among mean PAH concentrations according to the amount of coverage. Mains with > 0–50% coverage also had higher mean PAH levels in the flush samples (data not shown). These findings support the hypothesis that coating erosion may contribute to increased PAH levels. The year the main was installed was not a significant predictor of the level of coating coverage (data not shown).

*Human health risk*. We compared the PAH levels in the water samples to the drinking water quality standards of 0.1 µg/L for Σ10PAH and 0.01 µg/L for BaP (Drinking Water Decree 2011). Under normal operation (samples for undisturbed 1 and 2), we observed no exceedances of PAH standards (Table 2). However, PAH levels in some samples collected shortly after flushing and repair exceeded the Σ10PAH (29% and 40% of samples, respectively) and BaP (24% and 6%) standards, and 31% of samples collected 2 days after repair exceeded the Σ10PAH standard.

**Figure 4:**
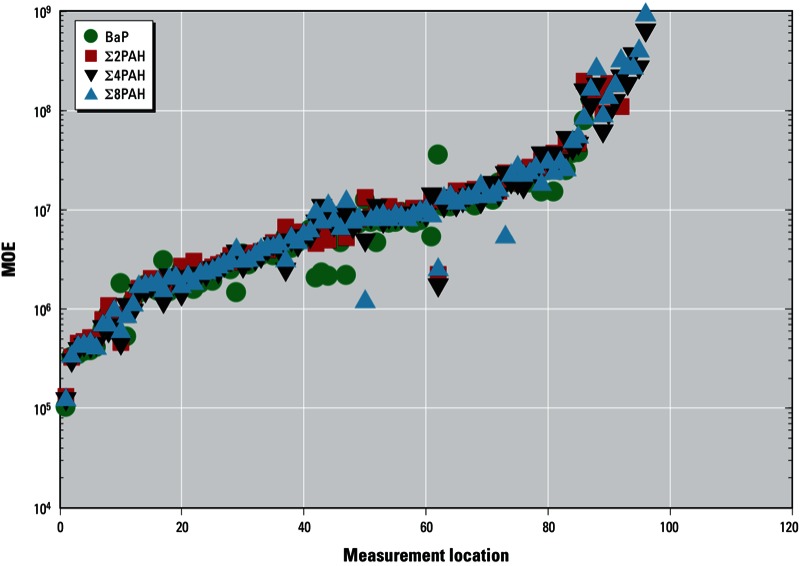
MOEs for exposure to carcinogenic PAHs (BaP, Σ2PAH, Σ4PAH, and Σ8PAH) through drinking water at all 120 measurement locations. MOEs for samples with no measurable PAHs are infinite and are therefore not shown. Annual oral intakes were estimated based on concentrations in the eight samples from each location after accounting for estimated exposure durations for each sample type.

An exposure with an MOE of ≥ 10,000 is considered to be of low concern for public health and low priority for risk management action (EFSA 2005). Based on the maximum Σ8PAH concentration measured in any sample of a given type, MOEs calculated for oral exposure in an adult weighing 60 kg and consuming 2 L of water per day were < 10,000 for low- and high-flush samples, after-flush samples (collected 15 min after flushing), and samples taken 2–4 hr after repair (Table 2). However, these MOEs were based on the highest measured level, and the test conditions resulting in MOEs < 10,000 are likely to be uncommon within a distribution network. Therefore, we calculated MOEs for carcinogenic effects at each individual location after estimating annual oral intakes based on the BaP, Σ2PAH, Σ4PAH, and Σ8PAH concentrations in the eight routinely collected samples from each location after accounting for estimated exposure durations for each sample type: 1 day of exposure to the PAH concentration measured in the low-flush sample, 1 day for the high-flush concentration, 2 days for after-flush, 2 days for repair 1, 40 days for repair 2, and 319 days of exposure to the maximum concentration measured in the three samples collected during undisturbed operation (Table 1). Because we included exposures based on concentrations in flush samples collected from hydrants (water which is not normally consumed), we considered these intake estimates to be conservative (i.e., higher than would be expected for most consumers). Using this method, all MOEs estimated for the 120 locations were > 10,000, with the lowest estimated MOE of 104,000 (Figure 4).

Estimates of inhalation exposure, derived using ConsExpo (RIVM 2010) and using the maximum concentration measured in any sample for each individual PAH, indicated that inhalation exposure by aerosol formation during showering was negligible compared with estimated oral intakes (< 1% of oral exposure for Σ16PAH; data not shown). However, inhalation exposure due to avaporation of volatile PAHs (2.0 µg/kg bw/day) was comparable to oral exposure (2.1 µg/kg bw/day) for Σ16PAH. However, only 0.2%, 10%, 11%, and 6.9% of the total exposure to the carcinogenic PAHs BaP, Σ2PAH, Σ4PAH, and Σ8PAH, respectively, were attributed to inhalation during showering, which suggests that inhalation exposure primarily involves PAHs classified as noncarcinogens or as low potency carcinogens. Therefore, we did not include inhalation exposure during showering when assessing risk for carcinogenic effects of PAHs in drinking water.

We determined that oral and inhalation exposures to the maximum concentration of Σ16PAH totaled 4.1 µg/kg bw/day (Table 4), whereas the lowest derived TDI for noncarcinogenic effects of exposure during a single day is 30 μg/kg bw/day (Baars et al. 2001). Thus, toxic effects other than carcinogenic effects are not likely to occur.

**Table 4:** Estimated oral and inhalation exposures to measured and summed PAHs.

PAH	Max conc (μg/L)	Exposure (μg/kg bw/day)	
		Inhaled	Oral
Acenaphtene	0.96	0.082	0.032
Acenaphtylene	0.64	0.055	0.021
Anthracene	1.7	0.14	0.057
Benz[a]anthracene	4.3	0.0093	0.14
Benzo[b]fluoroanthene	3.8	0.024	0.13
Benzo[k]fluoroanthene	2.2	4.8 × 10–6	0.073
Benzo[ghi]perylene	2.6	5.7 × 10–6	0.087
Benzo[a]pyrene	3.6	2.7 × 10–4	0.12
Chrysene	3.6	0.028	0.12
Dibenz[ah]anthracene	1.8	5.3 × 10–6	0.060
Fluoroanthene	11.0	0.32	0.37
Fluorene	1.4	0.12	0.047
Indeno[1,2,3-cd]pyrene	2.7	5.3 × 10–6	0.090
Naphtalene	7.4	0.63	0.25
Phenanthrene	6.0	0.51	0.20
Pyrene	6.6	0.16	0.22
Σ2PAH		0.028	0.24
Σ4PAH		0.061	0.51
Σ8PAH		0.061	0.82
Σ16PAH		2.1	2.0
Estimates are based on the maximum measured concentration (Max conc) of that PAH in any of the samples from the 120 locations. Summed PAHs are theoretical values because they include the the highest concentration measured for the individual PAH in any of the samples collected from all 120 locations (i.e., the highest concentration of the PAH in > 960 samples).			

*General discussion*. Exceedance of PAH standards can occur after invasive repairs in cast iron water mains, but our estimates suggest that human health risks related to coated cast iron water mains are low. To prevent increased exposures during flushing operations, drinking water networks should be flushed in a systematic way to ensure that all particles containing PAH are removed (Vreeburg 2010).

Water quality aspects, such as hardness (WHO 2003), chlorination (Maier D et al. 1997; Maier M et al. 2000b), and oxidation (Read and Whiteoak 2003), may affect PAH levels in drinking water. Chlorination is not used to disinfect drinking water in the Netherlands, and we did not evaluate hardness and oxidation, which have varied over time. We also did not assess the influence of biofilm on PAH levels, as previously suggested (Maier D et al. 1997; Maier M et al. 2000b).

PAH levels are likely to be increased in cast iron water mains as a consequence of invasive work and raised flow velocities in water distribution networks in coutries other than the Netherlands. However, the specific circumstances associated with elevated PAH levels may differ, for example, in countries where chlorine is used as a disinfectant, and additional field measurements would be needed to determine whether our findings apply elsewhere.

## Conclusions

PAHs from coal tar and bitumen coatings in cast iron water mains are a potential health risk for humans. Drinking water samples were collected from 120 measurement locations in cast iron water distribution networks in the Netherlands over a period of 17 days under various operational conditions: undisturbed operation, during flushing, and after repair of water mains. We analyzed these samples for PAHs and estimated the health risk using the MOE approach. During flushing, high PAH levels may occur, but PAH concentrations dropped rapidly after flushing. After coated cast iron mains were cut and repaired, we observed PAH levels that exceeded the water quality standard up to 40 days after the repair. However, even after accounting for such exceedances, estimated MOEs were > 10,000 for all 120 measurement locations. Therefore, we conclude that PAH exposure through Dutch drinking water is of low concern for consumer health.
